#  Shuni Virus in Cases of Neurologic Disease in Humans, South Africa

**DOI:** 10.3201/eid2702.191551

**Published:** 2021-02

**Authors:** Thopisang P. Motlou, Marietjie Venter

**Affiliations:** University of Pretoria, Pretoria, South Africa

**Keywords:** neurologic disease, orthobunyavirus, arthropod-borne viruses, RT-PCR, Shuni virus, South Africa, viruses

## Abstract

We describe Shuni virus (SHUV) detection in human neurologic disease cases in South Africa. SHUV RNA was identified in 5% of cerebrospinal fluid specimens collected during the arbovirus season from public sector hospitals. This finding suggests that SHUV may be a previously unrecognized cause of human neurologic infections in Africa.

Arthropod-borne viruses (arboviruses) warrant attention in the global health landscape because of their potential to cause widespread epidemics worldwide (*1*). Epizootics in animals may signal an increase in virus activity and predict potential missed human outbreaks, as shown for West Nile virus neurologic infections in horses (*2**–**4*) and humans (*5*) and Rift Valley fever associated with abortion storms in livestock and cases of febrile and neurologic disease (*6*) and miscarriages in humans (*7**,**8*).

Arboviruses of African origin are largely responsible for the recent expansion in geographic range of emerging viruses worldwide. These viruses have been associated with human illness and death in new regions in recent years but remain underreported in Africa (*9*). Cases of neurologic arbovirus infections are thought to be underreported in humans in South Africa, with ≈3% of cerebrospinal fluid (CSF) samples of neurologic infections in humans testing positive for West Nile virus (*7*). This raised the question as to whether other neglected zoonotic arboviruses are circulating in Africa that may potentially cause future outbreaks in new regions (*10*).

Shuni virus (SHUV) has recently been described as a cause of neurologic infections in horses in South Africa (*11*) and emerged as a cause of neurologic infections and birth defects in livestock in Israel (*12*). Before this study, there had been only 1 confirmed human SHUV case since 1966 (*13*). We used real-time reverse transcription PCR (rRT-PCR) to investigate whether SHUV is associated with unsolved neurologic cases in humans in South Africa by screening archived CSF samples collected for viral diagnosis from hospitalized patients during the arbovirus season in January–May 2017.

## The Study

We obtained archived CSF specimens from public sector hospitals across Gauteng Province, South Africa, through the National Health Laboratory Service, Tshwane Academic division, from patients who had neurologic signs and symptoms during January–May 2017. We grouped the CSF specimens into 4 categories based on age: age group 1 was children (<1–12 years of age); age group 2, adolescents (13–18 years of age); age group 3, adults (19–59 years of age); and age group 4, senior adults (>60 years of age). SHUV-positive cases were determined by an *Orthobunyavirus* genus-specific RT-PCR and confirmed using Sanger sequencing and phylogenetic analysis.

We extracted RNA from the CSF samples using the QIAamp Viral RNA Kit (QIAGEN, https://www.qiagen.com). We performed an *Orthobunyavirus* genus-specific RT-PCR using the Agpath-ID One Step RT-PCR (Thermo Fisher Scientific, https://www.thermofisher.com) with primers designed to amplify a 155-bp fragment of the nucleocapsid gene of the small (S) segment of orthobunyaviruses (*14*). We analyzed the sequences using the BioEdit DNA sequence alignment editor v7.0 5.3 software (*15*) and Blast search analysis (https://blast.ncbi.nlm.nih.gov/Blast.cgi). We performed phylogenetic analysis using maximum-likelihood analysis (MEGA X, http://www.megasoftware.net) as confirmation that the amplicons represent SHUV (Figure, panel A). A larger region of the S segment (≈460 bp) could be sequenced for only 1 of the positive samples (ZRUH131/17, GenBank accession no. MN937197) (Figure, panel B) because of low viral RNA concentration and sample volume in the other CSF samples.

**Figure:**
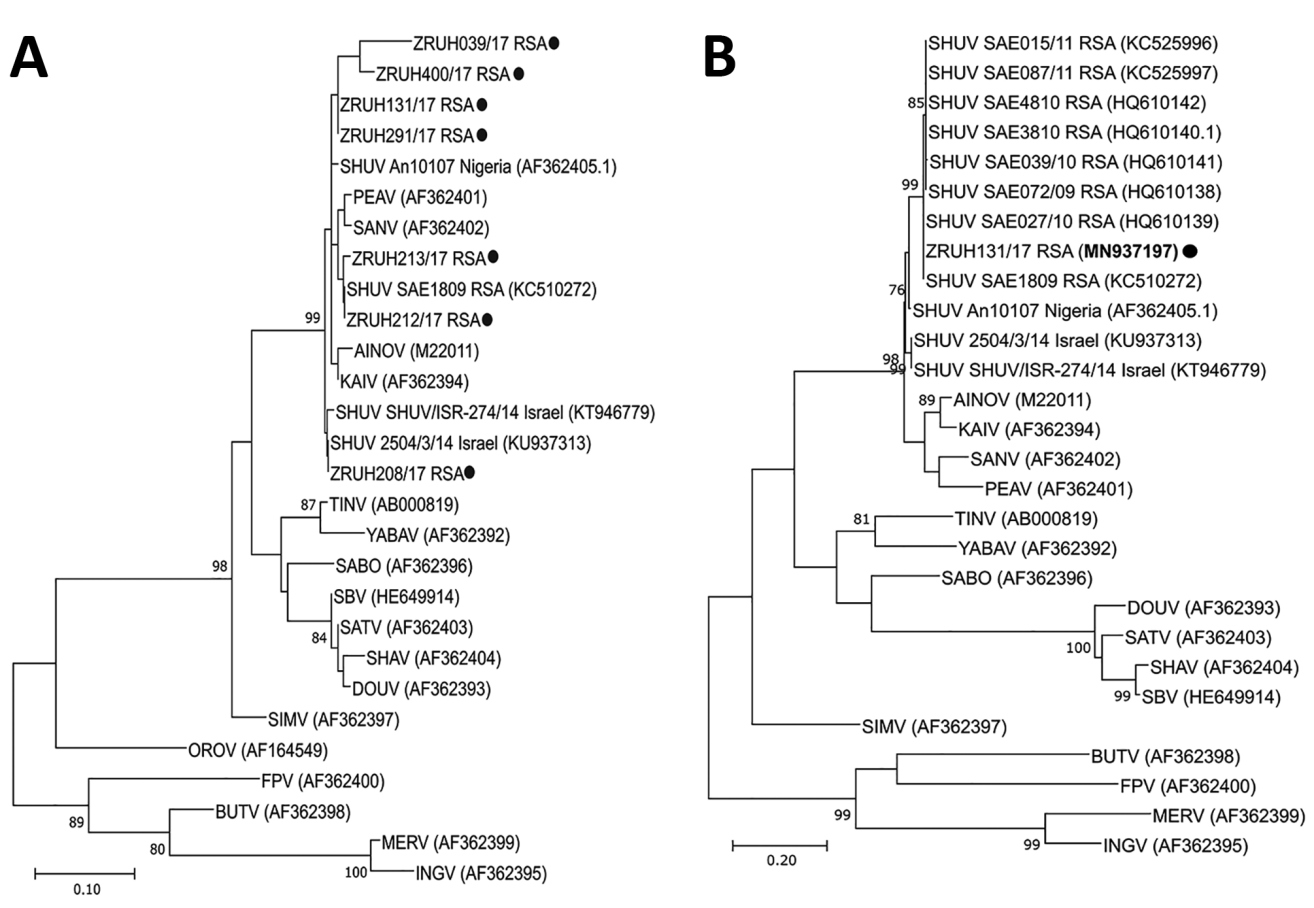
A) Phylogenetic confirmation that the orthobunyavirus small (S) segment specific reverse transcription PCR (*14*) positive products identified in this study clustered with SHUV strains. The 155-bp sequence of the nucleocapsid gene of the S segment of the human clinical isolates were aligned to SHUV strains previously identified in animals and other Orthobunyaviruses in the Simbu serogroup. The evolutionary history was inferred by using the maximum likelihood method and Kimura 2-parameter model. The tree with the highest log likelihood (−1043.27) is shown. The percentage of trees in which the associated taxa clustered together is shown next to the branches. Initial tree(s) for the heuristic search were obtained automatically by applying neighbor-joining and BioNJ algorithms to a matrix of pairwise distances estimated using the maximum composite likelihood (MCL) approach and then selecting the topology with superior log likelihood value. A discrete gamma distribution was used to model evolutionary rate differences among sites (5 categories [+G parameter = 0.6884]). This analysis involved 28 nt sequences. All positions containing gaps and missing data were eliminated (complete deletion option). There were a total of 151 positions in the final dataset. Evolutionary analyses were conducted in MEGA X (http://www.megasoftware.net). Black circles indicate the newly sequenced positive human samples (ZRUH208/17, ZRUH131/17, ZRUH219/17, ZRUH212/17, ZRUH213/17, ZRUH400/17, ZRUH039/17). B) Phylogenetic analysis of a human SHUV-positive case using a larger region of the S-segment amplified with SHUV-specific primers. The evolutionary history was inferred by using the maximum likelihood method and Tamura-Nei model. The tree with the highest log likelihood (−3135.73) is shown. The percentage of trees in which the associated taxa clustered together is shown next to the branches. Initial tree(s) for the heuristic search were obtained automatically by applying neighbor-joining and BioNJ algorithms to a matrix of pairwise distances estimated using the MCL approach and then selecting the topology with superior log likelihood value. A discrete gamma distribution was used to model evolutionary rate differences among sites (5 categories [+G, parameter = 0.3230]). This analysis involved 28 nt sequences. All positions containing gaps and missing data were eliminated (complete deletion option). There were a total of 324 positions in the final dataset. Evolutionary analyses were conducted in MEGA X. Black circle indicates the newly sequenced positive human strain (ZRUH131/17, GenBank accession no. MN937197). Sequence data are available upon request; numbers in parentheses for related strains indicate GenBank accession numbers. Scale bars indicate nucleotide substitutions per site. AINOV, Aino virus; AKAV, Akabane virus; BUTV, Buttonwillow virus; DOUV, Douglas virus; FPV, Faceys Paddock virus; INGV, Ingwavuma virus; KAIV, Kaikalur virus; KAIRV, Kairi virus; MERV, Mermet virus; OROV, Oropouche virus; PEAV, Peaton virus; SABOV, Sabo virus; SANV, Sango virus; SATV, Sathuperi virus; SBV, Schmallenburg virus; SHAV, Shamonda virus; SHUV, Shuni virus; SIMV, Simbu virus; TINV, Tinaroo virus; THIV, Thimiri virus; YABA, Yaba-7 virus.

A total of 7 of 130 (5.4%) CSF samples tested positive with an *Orthobunyavirus* rRT-PCR targeting the S segment and were confirmed by DNA sequencing to represent SHUV (Figure, panel A). A longer region was obtained for a CSF sample taken from a patient who was confirmed to have had neurologic diseases (meningitis, encephalitis, and seizures) with a clinical diagnosis of TB meningitis. Apart from neurological signs, additional clinical diagnoses in other patients included respiratory diseases (tuberculosis, upper respiratory tract infection, and pneumonia), gastrointestinal diseases, vomiting, and hydrops fetalis (Table 1). Only 3 patients’ HIV status was recorded, of which 1 patient’s mother was confirmed to be HIV positive and undergoing treatment. The baby of the positive mother subsequently received nevirapine. The other 2 patients were HIV negative; however, 1 of the children was given nevirapine for reasons not stated. No apparent travel history was recorded for any of these patients.

**Table 1:** Demographic and clinical information of SHUV-positive CSF samples from 7 patients hospitalized with neurologic signs, Gauteng Province, South Africa, 2017*

Sample ID	Patient age/sex	Other symptoms	Clinical diagnoses	HIV status	Other tests	Vaccination	Reason for discharge	Location
ZRUNH039/17	29 y/F	Not stated	Meningitis	Unknown	Not stated	Unknown	Unknown	JHB
ZRUNH131/17	1 y 9 mo/M	Not stated	TB, meningitis	Unknown	Not stated	Unknown	Unknown	JHB
ZRUNH219/17	6 mo/F	Vomiting, diarrhea, fine maculopapular rash	Acute gastroenteritis and shock	Mother (positive), on HAART/ PMTCT, ART (FDC); baby received nevirapine	*H. influenzae* Ag (negative), *N. meningitidis* ACV W135 (negative), *E. coli *(negative), *S. pneumonia *(negative), GBS (negative), cryptococcal Ag (negative)	Mother did not have clinic card	Stable	Eastlynne, Pretoria
ZRUNH212/17	2 y 8 mo/M	Coughing blood, otitis media, simple febrile seizures, fever (38°C), difficulty breathing, vomiting, diarrhea; had second episode of seizure	Upper respiratory tract infection/ hemoptysis/ febrile convulsions	Mother negative; baby received nevirapine	Not stated	Up to date: BGG, polio+DPT (3–18 mo), DT (5 y) not done	Stable	Pretoria
ZRUNH208/17	4 y 11 mo/M	Seizures, ICU patient, decreased LOC, vomiting, seizures, fever, diarrhea	Encephalitis and aspiration pneumonia	Negative	Microbiology: negative for bacteria	Incomplete: no polio+DPT (4,5 mo)	Not stated	Eastlynne, Pretoria
ZRUNH213/17	13 d/F	ICU patient, baby delivered normally, neonatal encephalopathy, second-degree congenital sepsis/TORCH, poor sucking, premature, low birthweight, nonimmune, subcutaneous edema, abdominal distension (HC, chest, AC), abdominal U/S (ascites, bilateral dense kidneys)	Nonimmune hydrops fetalis	Not stated	HSV (positive; patient tested negative following treatment), rubella PCR (IgG positive, IgM negative), CMV (IgG positive, IgM negative)	Up to date	Stable	Mamelodi East, Pretoria
ZRUNH400/17	4 mo/M	Respiratory distress, vomiting bile	Viral pneumonia	Not stated	Not stated	Up to date	Not stated	Olieven-houtbosch, Pretoria

*AC, abdominal circumference; Ag, antigen; BCG, bacille Calmette-Guérin; CMV, cytomegalovirus; DPT, diphtheria/pertussis/tetanus; *E. coli*, *Escherichia coli*; FDC, fixed-dose combination; GBS, group B *Streptococcus*; *H. influenzae*, *Haemophilus influenzae*; HAART, highly active antiretroviral therapy; HC, hepatitis C; HSV, herpes simplex virus; ICU, intensive care unit; ID, identification; JHB, Johannesburg; LOC, level of consciousness; *N. meningitis*, *Neisseria meningitidis*; PMTCT, prevention of mother-to-child transmission*; *SHUV, Shuni virus; TB, tuberculosis; TORCH, *Toxoplasma gondii*; U/S, ultrasound.

Most specimens screened were from children (63.1%). Groups with the lowest number of patients were adolescents (1.5%) and the elderly (4.6%) (Table 2). There was only a slight difference in the percentage of males and female patients tested (46.2% male and 51.7% female). A total of 6 (85.7%) of 7 positive cases were in children and 1 of 7 (14.3%) was in an adult. Three of the children with positive test results were <6 months of age. One of these positive children was a newborn admitted to the intensive care unit at 13 days of age who had not left the hospital since birth. Aside from neurologic signs that were present in all patients, the most common recorded symptoms were vomiting, diarrhea, seizures, and fever.

**Table 2:** Demographics of the patient group screened for Shuni virus (SHUV), Gauteng Province, South Africa, 2017

Demographic	Total no. (%)	No. (%) positive	Odds ratio (95% CI)
Total	130	7 (5.3)	

Age group, y*			
1	82 (63.1)	6 (4.9)	0.05333 (0.01951–0.1458)
2	2 (1.5)	0 (0)	0
3	40 (30.8)	0 (0)	0
4	6 (4.6)	1 (16.7)	0.2 (0.02337–1.71188)
Sex			
M	60 (46.2)	3 (42.9)†	
F	67 (51.7)	4 (57.1)†	
Not stated	3 (2.3)	0	

*The classification of the age groups is based on the neutral network using the FG-NET aging database and wavelets. Age group 1, children (<1–12 y); age group 2, adolescents (13–18 y); age group 3, adults (19–59 y); and age group 4, senior adults (>60 y). p value = 0.6621. †Percentages based on total no. positive cases.

SHUV was reported in horses with severe neurologic signs in South Africa during 2009–2012 (*11*), which prompted us to also investigate its occurrence in human cases. Screening of CSF specimens from hospitalized patients with neurologic signs around Gauteng Province in South Africa, where some of the equine cases were detected, suggests that up to 5.4% of unidentified neurologic human cases during the arbovirus season may be caused by SHUV. Six of the 7 patients who tested positive for SHUV were children <5 years of age, with 1 being a newborn 13 days of age; only 1 case was identified in a woman. Three of the 7 patients were discharged after being found to be stable; the outcomes of the other 4 are unknown.

These patients were also tested for other viral and bacterial infections, such as influenza, *Neisseria meningitidis*, pneumonia, herpes simplex virus (HSV), rubella, and cytomegalovirus (Table 1). All 7 patients showed negative results for all requested diagnostic assays except for the 13-day-old infant, who received a diagnosis of hydrops fetalis. He was IgG positive for rubella and cytomegalovirus but IgM negative for both, suggesting maternal antibody transmission. The patient was positive for HSV by PCR and was subsequently placed on treatment for 12 days postnatal until the HSV PCR yielded a negative result. Although the diagnosis of a HSV co-infection cannot rule out HSV as the cause of the hydrops fetalis, the fact that he had not left the hospital since birth suggests a likely vertical transmission of both HSV and SHUV. The patient was stable at discharge after 21; with no death has been reported. None of the patients had any travel history, indicating that they may have been infected in or around their area of residence. Equine cases had previously been identified in these areas, suggesting possible similar vector exposure (*11*).

A limitation of this study was that all other potential causes of neurologic signs were not exhaustively investigated. Previous detection of SHUV in *Cullicoides* midges and *Culex theileri* mosquitoes (McIntosh BM, Epidemiology of arthropod-borne viruses in southern Africa. Unpublished thesis, University of Pretoria, 1980) suggests that SHUV has the potential to expand its geographic range and potentially emerge in new regions. The reservoir host for SHUV is not known but is thought to be ruminants and wildlife, from which transmission to humans would likely be accomplished through susceptible mosquitoes. 

## Conclusions

Detection of SHUV RNA in the CSF is highly suggestive of SHUV contributing to neurologic signs and likely crossing of the blood–brain barrier. However, further investigations with larger cohorts are needed to determine the disease burden of SHUV in humans across all age groups. These investigations can also include determining the geographic range, clinical presentation, potential vectors, and reservoir hosts in Africa. Improved diagnoses that include IgM serology and early PCR detection of SHUV will aid in defining the true incidence and epidemiology of SHUV.
